# Immunohistochemical study of BAX in the pyramidal layer of the hippocampus of rats in chronic cerebrovascular disorders modeling

**DOI:** 10.1002/alz70855_099594

**Published:** 2025-12-23

**Authors:** Dmitry Mednikov, Alexey Vladimirovich Smirnov, Ivan Nikolaevich Tyurenkov

**Affiliations:** ^1^ Volgograd State Medical University, Volgograd, Russian Federation; ^2^ Volgograd Medical Research Center, Volgograd, Russian Federation

## Abstract

**Background:**

Bax is a member of the pro‐apoptotic bcl‐2 subfamily and one of the major regulators of the intrinsic apoptotic pathway. Upon apoptotic stimuli, it is activated and oligomerizes on the mitochondrial outer membrane to mediate its permeabilization, which is considered a key step in apoptosis. Under physiological conditions, bax is largely cytosolic due to constant translocation from mitochondria to the cytosol by bcl‐xl, thereby avoiding the accumulation of toxic levels of bax on the mitochondrial outer membrane.

**Method:**

Chronic cerebral ischemia modeled in rats by limiting cerebral blood flow with applying ligatures to the carotid arteries for 28 days. Immunohistochemical study was performed using polyclonal antibodies to bax, visualized using diaminobenzidine.

**Result:**

Immunohistochemical study using antibodies against bax revealed uniform, predominantly weak cytoplasmic expression of bax‐positive material in all hippocampal zones. A reliable increase in the relative area of IM by 0.7% compared to the control was found in the CA1 zone (*p* <0.001). At the same time, the intensity of expression visually changed to moderately expressed, in individual neurons with signs of damage, expression of up to 3 points was determined.

**Conclusion:**

The significant increase in the relative area of bax‐positive material by 0.7% (*p* <0.001) in the CA1 hippocampus of rats with simulated carotid stenosis indicates, in our opinion, the involvement of bax in the implementation of irreversible damage to pyramidal neurons and regulated cell death in this zone.

